# Molecular Weight Distribution and Antioxidant Activity of Enzymatic Hydrolysates from *Rhopilema hispidum* and *Nemopilema nomurai* Under Different Enzymatic Hydrolysis Conditions

**DOI:** 10.3390/md23120447

**Published:** 2025-11-21

**Authors:** Xiaoxiao Liu, Lichao Teng, Wen Shen, Rongfeng Li, Song Liu, Ronge Xing, Huahua Yu

**Affiliations:** 1 Laboratory of Experimental Marine Biology, Institute of Oceanology, Chinese Academy of Sciences, Qingdao 266000, China; 2 Laboratory for Marine Drugs and Bioproducts, Qingdao Marine Science and Technology Center, Qingdao 266237, China; 3 University of Chinese Academy of Sciences, Beijing 100049, China

**Keywords:** jellyfish, *Rhopilema hispidum*, *Nemopilema nomurai*, enzymatic hydrolysate, molecular weight distribution, antioxidant activity

## Abstract

Jellyfish, as high-biomass marine resources, frequently exhibit explosive proliferation in coastal waters and possess both nutritional functional factors and potential medicinal value. This study investigated the enzymatic hydrolysis of two jellyfish species, *Rhopilema hispidum* (*R. hispidum*) and *Nemopilema nomurai* (*N. nomurai*), using Alcalase, Flavourzyme, and Protamex, with a specific focus on the molecular weight distribution and antioxidant activity. The optimal hydrolysis conditions were systematically determined via single-factor experiments followed by orthogonal test optimization. The Flavourzyme hydrolysates had the highest proportion of low-molecular-weight peptides (<3 kDa) and exhibited the most potent antioxidant activity, while Protamex hydrolysates had more high-molecular-weight peptides (>3 kDa, >40%) with comparatively weaker antioxidant activity. *R. hispidum* hydrolysates exhibited stronger DPPH• and O_2_•^−^ scavenging activities and contained a higher proportion of low-molecular-weight peptides (<3 kDa), whereas *N. nomurai* hydrolysates showed a higher degree of hydrolysis, and its hydrolysates demonstrated superior •OH scavenging activity. Chromatographic analysis confirmed low-molecular-weight peptides positively correlated with antioxidant potential. This study highlights molecular weight distribution, together with enzyme type, as a pivotal determinant of the antioxidant activity of jellyfish hydrolysates, providing insights for antioxidant peptide development.

## 1. Introduction

Jellyfish, as important plankton, are characterized by high species diversity and wide distribution. In recent years, influenced by factors such as global warming and eutrophication, the population of some jellyfish species has exhibited a trend of frequent and widespread blooms in coastal waters globally. Jellyfish blooms are often perceived as ecological nuisances due to their potential impacts on fisheries, tourism, and coastal infrastructure, while simultaneously providing new opportunities for the exploitation and utilization of jellyfish resources. Jellyfish are rich in a range of bioactive substances, with proteins being the primary organic component. These proteins are high-quality raw materials for peptide production. Owing to their low antigenicity, excellent biological safety, and minimal religious restrictions, they have gradually emerged as a subject of growing scientific interest.

Enzymatic hydrolysis serves as a prevalent method for jellyfish-derived peptide production due to its mild reaction conditions and controllable processes. Despite interspecies variations in enzymatic characteristics and bioactivities among enzymatic hydrolysates, different enzymatic hydrolysates may exhibit the same activity, as documented in neutral protease hydrolysates of *Rhopilema esculentum* [1], multi-enzyme hydrolysates of *Lobonema smithii* [2,3], pepsin/collagenase hydrolysates of four Mediterranean jellyfish species [4,5], and pepsin hydrolysates of *R. hispidum* [6]. In addition, functional divergence manifests among protease-specific hydrolysates within individual species. The neutral protease hydrolysates of *Rhopilema esculentum* demonstrate angiotensin-converting enzyme (ACE) inhibitory activity [1], whereas its triple-enzyme hydrolysates accelerate wound healing [7]. Similarly, the flavourzyme hydrolysates of *Lobonema smithii* exhibit immunomodulatory and tyrosinase-inhibitory effects [2], while its papain hydrolysates possess anti-inflammatory and antimicrobial activities [8]. These findings collectively indicate that enzyme selection plays a critical role in determining the functional properties of the resulting hydrolysates. Molecular weight distribution is a crucial factor influencing the activity of enzymatic hydrolysates. However, most current studies on jellyfish enzymatic hydrolysates focus on their activity, with insufficient research on the molecular weight distribution and difference in activity of hydrolysates prepared under different enzymatic hydrolysis conditions. In addition, at present, research on jellyfish-derived peptides has mainly focuses on *Rhopilema esculentum* and common jellyfish species in the Mediterranean Sea [9,10], while the research on *R. hispidum* and *N. nomurai* remains scarce. Considering the differences in proteins among different jellyfish species and growth environments, which in turn lead to variations in their enzymatic hydrolysates, coupled with the frequent explosive proliferation of *R. hispidum* and *N. nomurai* in East Asian, conducting research on their high-value utilization is an urgent practical need.

Excessive reactive oxygen species (ROS) can elicit a spectrum of oxidative stress responses, thereby inducing cellular damage, senescence, and the initiation and progression of multiple diseases such as cancers, neurodegenerative disorders, and cardiovascular diseases [11]. As a potential source of natural antioxidants, jellyfish-derived peptides have garnered increasing attention in research and development. This study aims to investigate the molecular weight distribution and antioxidant activity of *R. hispidum* and *N. nomurai* enzymatic hydrolysates under different enzymatic hydrolysis conditions, including optimizing the enzymatic hydrolysis process to enhance peptide yield, and comparing the molecular weight distribution and antioxidant activity of the hydrolysates, thereby providing valuable insights for high-value utilization of jellyfish resources in applications of functional foods, cosmetics, and pharmaceuticals.

## 2. Results and Discussion

### 2.1. Single-Factor Experiment

The bioactivity of hydrolysate depends on both substrate proteins and hydrolysis parameters, with the latter directly regulating the degree of hydrolysis (DH) to influence hydrolysate characteristics. Different enzymatic conditions generate peptide fragments with varying molecular weight distributions and amino acid compositions, which in turn lead to differences in bioactivity [12]. Enzyme dosage, time, temperature, pH, and liquid-to-solid ratio are critical parameters affecting enzyme activity and hydrolysis efficiency. This study employed single-factor experiments, sequentially varying one parameter while fixing the other four, to screen optimal hydrolysis conditions for Alcalase, Flavourzyme, and Protamex.

**Figure 1:**
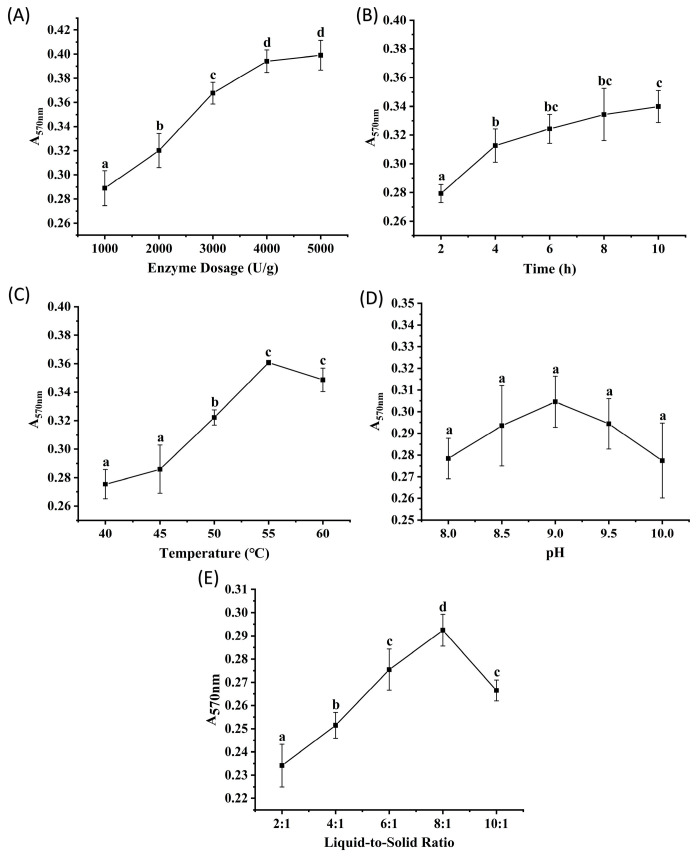
The results of single factor experiments of Alcalase hydrolysis. (**A**) enzyme dosage, (**B**) time, (**C**) temperature, (**D**) pH, (**E**) liquid-to-solid ratio. Different lowercase letters denote significant differences at *p* < 0.05.

Alcalase, derived from the fermentation of *Bacillus licheniformis*, is known for its high efficiency in protein degradation [13]. Figure 1A–E show the effects of enzyme dosage, hydrolysis time, temperature, pH, and liquid-to-solid ratio on the peptide content of the hydrolysates, determined by measuring absorbance. The results showed that absorbance increased significantly (*p* < 0.05) as the enzyme dosage increased from 1000 to 4000 U/g, with no significant difference observed between 4000 and 5000 U/g (*p* > 0.05). This suggests that enzyme-substrate binding reached saturation at 4000 U/g. Absorbance plateaued after 6 h of hydrolysis (no significant change between 6 to 10 h, *p* > 0.05), indicating that the reaction reached equilibrium at this time point. Within the temperature range of 40 to 60 °C, the groups at 55 to 60 °C exhibited significantly higher absorbance than those at lower temperatures (*p* < 0.05), likely due to the enhanced catalytic activity of the enzyme at elevated temperatures. No significant difference in hydrolysis efficiency was detected across the pH range of 8.0 to 10.0 (*p* > 0.05). A liquid-to-solid ratio of 8:1 resulted in significantly higher absorbance compared to other tested ratios (*p* < 0.05). Based on these results, orthogonal array experiments were designed with three levels for each of the following factors: enzyme dosages of 2000, 3000, and 4000 U/g; hydrolysis times of 4, 6, and 8 h; temperatures of 50, 55, and 60 °C; pH values of 8.5, 9.0, and 9.5; and liquid-to-solid ratios of 6:1, 8:1, and 10:1 (*v/m*).

**Figure 2:**
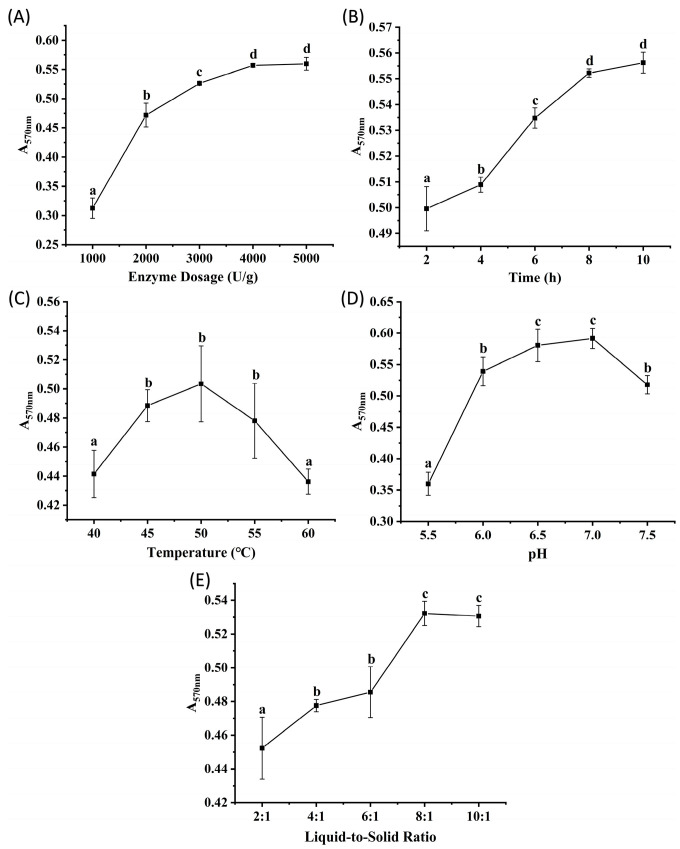
The results of single factor experiments of Flavourzyme hydrolysis. (**A**) enzyme dosage, (**B**) time, (**C**) temperature, (**D**) pH, (**E**) liquid-to-solid ratio. Different lowercase letters denote significant differences at *p* < 0.05.

Flavourzyme, derived from *Aspergillus oryzae*, is an enzyme commonly used in the food industry due to its ability to mitigate the formation of bitter peptides during protein hydrolysis [14]. Figure 2A–E show the effects of enzyme dosage, hydrolysis time, temperature, pH, and liquid-to-solid ratio on the peptide content of the hydrolysates, measured by absorbance. The results showed trends similar to those observed with Alcalase. Absorbance increased significantly (*p* < 0.05) as the enzyme dosage increased from 1000 to 4000 U/g but plateaued between 4000 and 5000 U/g (*p* > 0.05), suggesting that enzyme-substrate binding reached saturation at 4000 U/g. Absorbance stabilized after 8 h of hydrolysis (no significant change from 8 to 10 h, *p* > 0.05), indicating that the reaction reached equilibrium. Within the temperature range of 40 to 60 °C, groups incubated at 45, 50, and 55 °C exhibited significantly higher absorbance than those at other temperatures (*p* < 0.05), possibly because elevated temperatures enhance enzymatic activity. However, the notable decrease at 60 °C suggested thermal denaturation of the enzyme. The optimal pH range for Flavourzyme was determined to be 6.5 to 7.0, as these conditions resulted in significantly higher absorbance values than other pH (*p* < 0.05). Liquid-to-solid ratios of 8:1 and 10:1 resulted in significantly higher peptide yields than other ratios (*p* < 0.05). Based on these results, orthogonal array experiments were designed with the following parameter ranges: enzyme dosages of 2000, 3000, and 4000 U/g; hydrolysis times of 4, 6, and 8 h; temperatures of 45, 50, and 55 °C; pH values of 6.5, 7.0, and 7.5; and liquid-to-solid ratios of 6:1, 8:1, and 10:1 (*v/m*).

**Figure 3:**
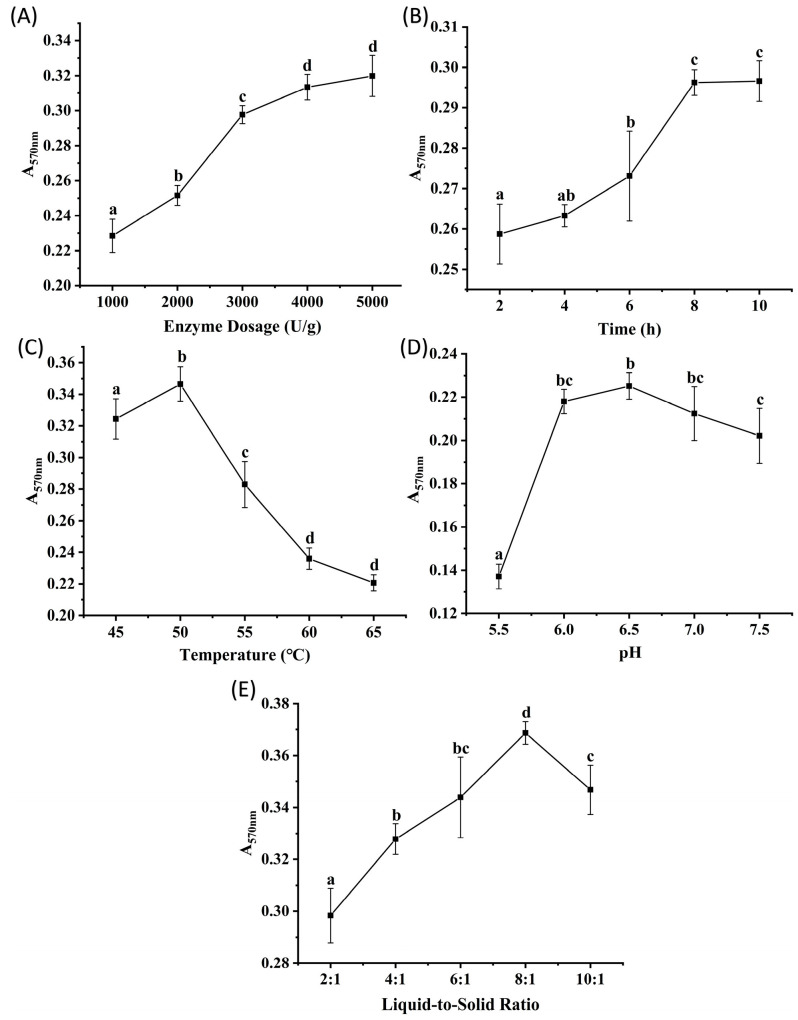
The results of single factor experiments of Protamex hydrolysis. (**A**) enzyme dosage, (**B**) time, (**C**) temperature, (**D**) pH, (**E**) liquid-to-solid ratio. Different lowercase letters denote significant differences at *p* < 0.05.

Protamex, derived from the fermentation of *Bacillus subtilis*, is characterized by its broad specificity for peptide bonds, neutral pH activity range, and ease of availability [15]. Figure 3A–E show the effects of enzyme dosage, hydrolysis time, temperature, pH, and liquid-to-solid ratio on the peptide content of the hydrolysates, determined by absorbance measurements. The results revealed trends consistent with those observed for Alcalase and Flavourzyme. Absorbance increased significantly (*p* < 0.05) as the enzyme dosage increased from 1000 to 4000 U/g, with no significant difference observed between 4000 and 5000 U/g (*p* > 0.05), suggesting saturation of enzyme-substrate binding at 4000 U/g. Absorbance plateaued at 8 h of hydrolysis (no significant change between 8 and 10 h, *p* > 0.05) and was significantly higher than at other time points (*p* < 0.05), indicating that 8 h was sufficient to reach reaction equilibrium. The temperature of 50 °C resulted in significantly higher absorbance than those at other temperatures tested (*p* < 0.05). The absorbance observed no significant differences (*p* > 0.05) at pH values of 6.0, 6.5, and 7.0, suggesting an optimal pH range of 6.0 to 7.0 for Protamex. A significantly higher absorbance was observed at the liquid-to-solid ratio of 8:1 compared to other ratios (*p* < 0.05). Based on these results, orthogonal array experiments were designed with three levels for each of the following factors: enzyme dosages of 2000, 3000, and 4000 U/g; hydrolysis times of 4, 6, and 8 h; temperatures of 45, 50, and 55 °C; pH values of 6.0, 6.5, and 7.0; and liquid-to-solid ratios of 6:1, 8:1, and 10:1 (*v/m*).

### 2.2. Orthogonal Experiment

Based on the optimal hydrolysis parameter ranges identified from single-factor experiments, an L_27_ (3^5^) orthogonal design was adopted to further optimize the hydrolysis conditions for each enzyme. Five factors including time, liquid-to-solid ratio, temperature, enzyme dosage, and pH were selected to systematically screen out the optimal single-enzyme process for jellyfish peptide preparation.

For the orthogonal experiment results of Alcalase (Table 1), the R-value characterizes the influence intensity of each factor on hydrolysis efficiency, and the order of their main effects is enzyme dosage (U/g) > time (h) > temperature (°C) > liquid-to-solid ratio (*v/m*) > pH. ANOVA analysis confirmed that all five factors significantly affected the peptide content in the enzymatic hydrolysate (*p* < 0.05). The optimal process combination determined by k-value optimization was A_3_B_1_C_3_D_1_E_3_, corresponding to a temperature of 60 °C, a time of 8 h, a liquid-to-solid ratio of 6:1, an enzyme dosage of 2000 U/g, and a pH of 9.5.

For the orthogonal experiment results of Flavourzyme (Table 2), the R-value (range) characterizes the influence intensity of each factor on hydrolysis efficiency. The order of main effects is enzyme dosage (U/g) > liquid-to-solid ratio (*v/m*) > temperature (°C) > time (h) > pH. ANOVA analysis showed that enzyme dosage, time, liquid-to-solid ratio, and temperature had significant effects on the hydrolysis process (*p* < 0.05), while pH had no significant effect (*p* > 0.05). The optimal combination determined by k-value optimization was A_2_B_3_C_3_D_3_E_2_, corresponding to a temperature of 50 °C, a time of 6 h, a liquid-to-solid ratio of 10:1 (*v/m*), an enzyme dosage of 3000 U/g, and a pH of 7.0.

For the orthogonal experiment results of Protamex (Table 3), the R-value (range) characterizes the influence intensity of each factor on hydrolysis efficiency. The order of main effects is time (h) > enzyme dosage (U/g) > pH > temperature (°C) > liquid-to-solid ratio (*v/m*). ANOVA analysis showed that enzyme dosage, time, temperature, and pH had significant effects on the hydrolysis process (*p* < 0.05), while the liquid-to-solid ratio had no significant effect (*p* > 0.05). The optimal combination determined by k-value optimization was A_3_B_3_C_2_D_3_E_3_, corresponding to a temperature of 50 °C, a time of 8 h, a liquid-to-solid ratio of 10:1 (*v/m*), an enzyme dosage of 4000 U/g, and a pH of 7.0.

The optimized enzymatic hydrolysis conditions obtained from orthogonal experiments are summarized in Table 4. Considering that a relatively high liquid-to-solid ratio in practical applications would lead to time-consuming and economic costs for peptide preparation, the liquid-to-solid ratio was uniformly adjusted to 6:1 (*v/m*) with reference to the results of single-factor and orthogonal experiments.

### 2.3. Degree of Hydrolysis (DH)

**Figure 4:**
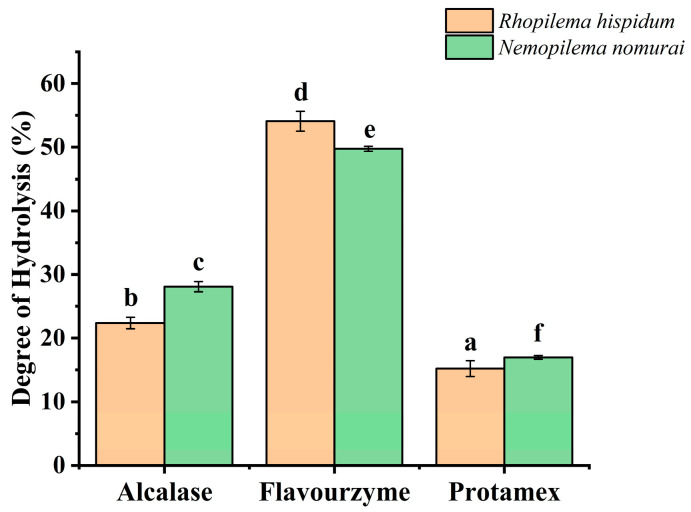
Effect of different enzymes on degree of hydrolysis. (Different lowercase letters indicate significant differences (*p* < 0.05) among different jellyfish species treated with different enzymes).

Significant differences (*p* < 0.05) in degree of hydrolysis (DH) were observed among the hydrolysates produced by Alcalase, Flavourzyme, and Protamex, with DH ranging from 15.2% to 54.1% (Figure 4). DH characterizes the efficiency of peptide bond cleavage during protein enzymatic hydrolysis and reflects the effect of enzyme activity on the substrate [16]. Among them, the DH of Flavourzyme was significantly higher than that of Alcalase and Protamex (*p* < 0.05), which may be attributed to its combined endopeptidase and exopeptidase activities that synergistically promote the production of free peptides and amino acids [17]. This observation is consistent with the enzymatic hydrolysis pattern reported by Maytamart Upata et al. for the jellyfish *Lobonema smithii* [2]. In contrast, both Alcalase and Protamex are dominated by endopeptidase activity, specifically targeting peptide bonds of neutral residues for hydrolysis [2].

It is noteworthy that the DH of *N. nomurai* hydrolyzed by Alcalase and Protamex was significantly higher than that of *R. hispidum* (*p* < 0.05), while the DH of *R. hispidum* hydrolyzed by Flavourzyme was significantly higher than that of *N. nomurai* (*p* < 0.05). This indicates that Flavourzyme has higher hydrolysis efficiency for *R. hispidum* protein, whereas Alcalase and Protamex are more suitable for hydrolyzing *N. nomurai* protein.

### 2.4. Molecular Weight Distribution

Studies have shown that molecular weight distribution affects the bioactivities of peptides, such as antioxidant activity [18], angiotensin-converting enzyme (ACE) inhibitory activity [19], skin-whitening activity [20], and immunomodulatory activity [21]. In general, antioxidant peptides are composed of 2 to 20 amino acids, with molecular weights usually less than 3 kDa [22,23]; ACE inhibitory peptides have a molecular weight range of 301 to 2037.26 Da; low-molecular-weight peptides also exhibit more significant skin-whitening and immunomodulatory activities [19]. It can thus be seen that a molecular weight below 3 kDa may be a critical threshold for significant peptide activity.

To investigate the molecular weight distribution of jellyfish hydrolysates, a calibration curve equation was established to relate retention time (Rt) to the logarithm of molecular weight (lg MW):

lg MW = −0.1399Rt + 5.4395

**Figure 5:**
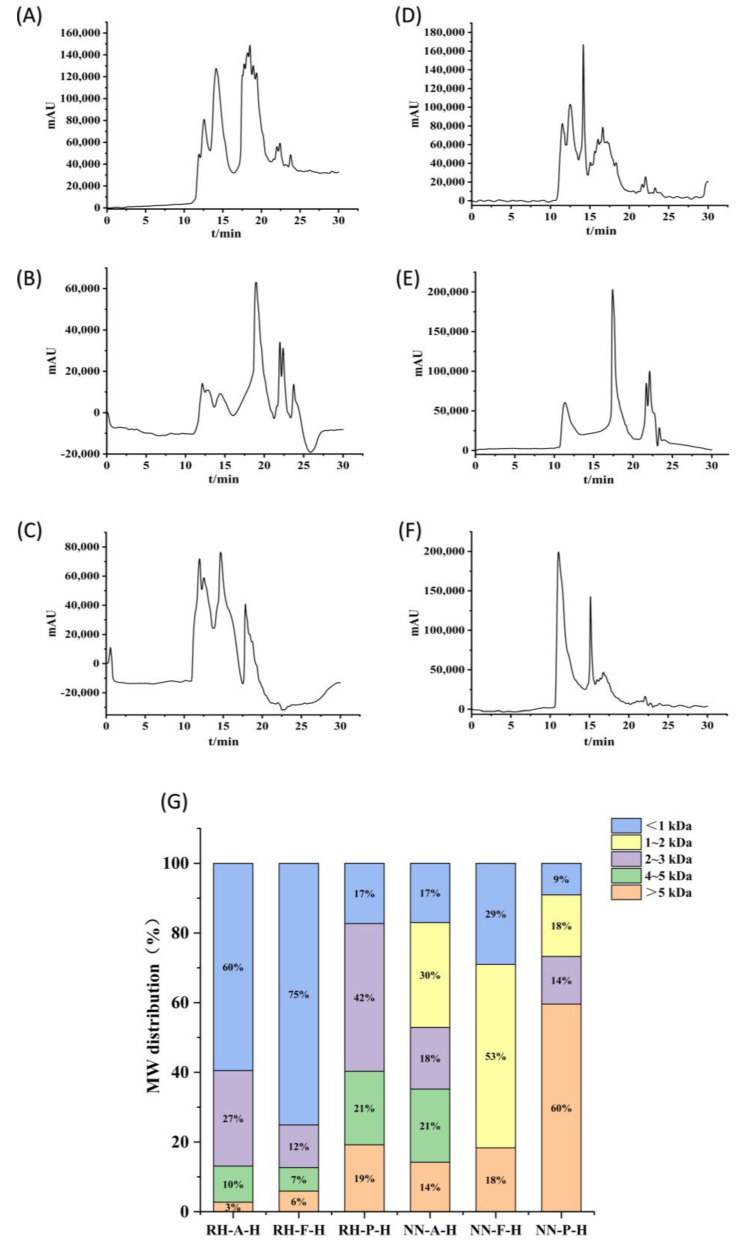
HPLC chromatograms of hydrolysates. (**A**) *R*. *hispidum* Alcalase hydrolysate (RH-A-H), (**B**) *R*. *hispidum* Flavourzyme hydrolysate (RH-F-H), (**C**) *R*. *hispidum* Protamex hydrolysate (RH-P-H), (**D**) *N. nomurai* Alcalase hydrolysate (NN-A-H), (**E**) *N. nomurai* Flavourzyme hydrolysate (NN-F-H), (**F**) *N. nomurai* Protamex hydrolysate (NN-P-H), (**G**) Molecular weight distribution of six hydrolysate.

The coefficient of determination (R^2^) was 0.9978, indicating a good linear relationship for compounds with molecular weights ranging from 100 to 6000 Da. Thus, the molecular weights of samples could be analyzed using this linear regression equation. The liquid chromatography results and molecular weight distributions of the samples are shown in Figure 5. In addition, the chromatograms (Figure 5A–F) revealed distinct elution profiles among the enzymatic treatments. Flavourzyme hydrolysates exhibited stronger peaks in the later retention-time region (larger Rt), indicating an increased abundance of low-molecular-weight peptides, whereas Protamex hydrolysates showed relatively pronounced early elution peaks (smaller Rt), corresponding to higher proportions of high-molecular-weight fractions and less extensive hydrolysis.

Among the three enzymes, Flavourzyme achieved the most efficient hydrolysis, yielding 87.3% and 81.7% of peptides with molecular weights < 3 kDa in *R. hispidum* and *N. nomurai* hydrolysates, respectively. In contrast, Protamex showed the weakest hydrolytic efficiency, with corresponding proportions of 59.7% and 40.5%, respectively. These results are consistent with the degree of hydrolysis. These findings suggest that Flavourzyme, which has both endo- and exopeptidase activities, can more effectively cleave protein substrates into shorter peptide chains compared to Alcalase and Protamex. Consequently, Flavourzyme hydrolysates display larger signal contributions at longer retention times (smaller MW region), resulting in a higher proportion of peptides below 3 kDa (Figure 5G).

In horizontal comparison, the proportion of peptides with molecular weights less than 3 kDa in *R. hispidum* hydrolysates was higher than that in *N. nomurai* hydrolysates, which may indicate that *R. hispidum* proteins are more easily enzymatically hydrolyzed into small peptides. Such a difference may be related to the structural characteristics of the two jellyfish species. The predominance of low-molecular-weight peptides (<3 kDa) is also noteworthy because these peptides often exhibit superior antioxidant activity due to their small size and enhanced diffusion ability. This observation is supported by the study of Stefania De Domenico et al., who reported that the antioxidant activity of *Rhizostoma pulmo* hydrolysates was inversely correlated with molecular weight, with the fraction below 3 kDa demonstrating the strongest activity [4]. Therefore, the higher proportion of small peptides in the Flavourzyme hydrolysates implies a potentially stronger biological activity, which will be further verified in subsequent sections.

### 2.5. Antioxidant Activity

**Figure 6:**
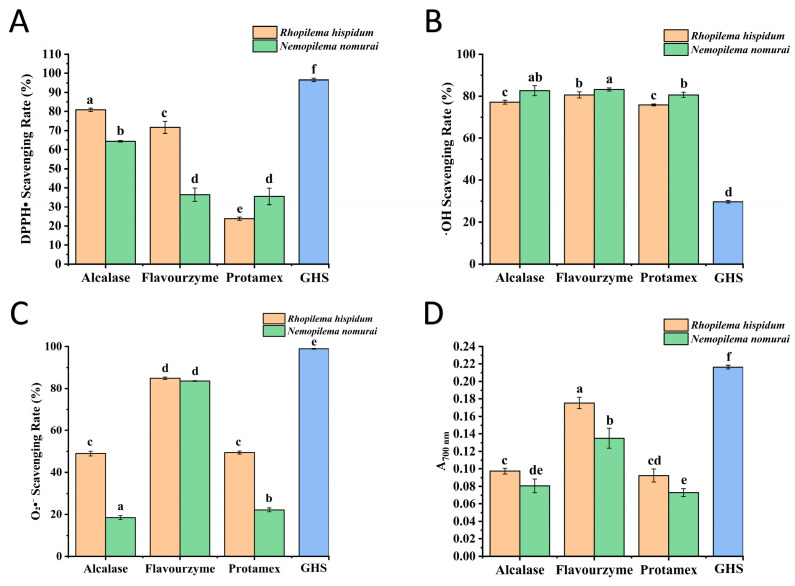
Determination of antioxidant activity. (**A**) Effect of different hydrolysates on DPPH radical scavenging activity, (**B**) Effect of different hydrolysates on •OH scavenging activity, (**C**) Effect of different hydrolysates on O_2_•^−^ scavenging activity, (**D**) Effect of different hydrolysates on reducing power, The GHS dataset has been scaled down to 10% of the original raw data. Different lowercase letters indicate significant differences (*p* < 0.05) among different jellyfish species treated with different enzymes.

Antioxidants terminate oxidative chain reactions by scavenging free radicals to form stable products. The 1,1-diphenyl-2-picrylhydrazyl radical (DPPH•) is a stable free radical widely used to evaluate the antioxidant activity of peptides, with a characteristic absorption peak at 517 nm. Antioxidants can reduce DPPH•, causing the characteristic purple color of its ethanol solution to fade. Therefore, the rate of change in absorbance can quantitatively characterize the scavenging capacity [24]. As shown in Figure 6A, there were significant differences in the DPPH• scavenging rates of hydrolysates from different enzymes for *R. hispidum*, with the order being Alcalase > Flavourzyme > Protamex (*p* < 0.05). For *N. nomurai*, no significant difference was observed between the hydrolysates of Flavourzyme and Protamex (*p* > 0.05). It is noteworthy that the scavenging rates of Alcalase hydrolysates from both jellyfish species were significantly higher than those of Flavourzyme and Protamex hydrolysates (*p* < 0.05), which is consistent with the conclusions of previous studies on marine biological peptides [25].

Hydroxyl radicals (•OH), the most reactive free radicals, can be generated from superoxide anions and hydrogen peroxide in the presence of metal ions such as iron and copper [26]. As shown in Figure 6B, hydrolysates exhibited strong •OH scavenging capacity with a scavenging rate exceeding 75%. The type of enzyme had no significant effect on the •OH scavenging rate of hydrolysates from the same jellyfish species. For *R*. *hispidum*, there was no significant difference between the hydrolysates of Alcalase and Protamex (*p* > 0.05). For *N. nomurai*, no significant difference was observed among the hydrolysates of Alcalase, Flavourzyme, and Protamex (*p* > 0.05). However, the •OH scavenging activity of *N. nomurai* hydrolysates was significantly higher than that of *R*. *hispidum* hydrolysates (*p* < 0.05). In both jellyfish species, the hydrolysates of Flavourzyme showed the highest •OH scavenging activity, which may be attributed to the fact that Flavourzyme has both endopeptidase and exopeptidase activities, resulting in hydrolysates with lower molecular weights [17].

The Superoxide Anion (O_2_•^−^) is a radical anion formed by the single-electron reduction in molecular oxygen. It is ubiquitous in aerobic cells and acts as a key mediator of oxygen toxicity [27]. As shown in Figure 6C, among the hydrolysates of the three enzymes, the O_2_•^−^ scavenging capacity of hydrolysates hydrolyzed by Flavourzyme was significantly higher than that by Alcalase and Protamex (*p* < 0.05), which may be attributed to their lower molecular weight distribution. Moreover, the O_2_•^−^ scavenging activity of *R. hispidum* hydrolysates was superior to that of *N. nomurai* hydrolysates. This result aligns with the characteristics of molecular weight distribution, indicating a negative correlation between scavenging activity and molecular weight: the lower the molecular weight distribution, the stronger the O_2_•^−^ scavenging capacity.

The reducing power is a key indicator for evaluating the antioxidant activity, which is commonly quantified using the ferric ion reducing antioxidant power (FRAP) assay [28]. This method is based on the ability of samples to reduce Fe^3+^ to Fe^2+^, and the determination is achieved by measuring the absorbance of the Prussian blue complex at 700 nm (positively correlated with reducing power). The specific mechanism is as follows: the reducing groups in samples reduce potassium ferricyanide (K_3_[Fe (CN)_6_]) to potassium ferrocyanide (K_4_[Fe(CN)_6_]), and the latter reacts with Fe^3+^ to form Fe_4_[Fe(CN)_6_]_3_ (blue). As shown in Figure 6D, hydrolysates hydrolyzed by Flavourzyme exhibited significantly higher reducing power than those hydrolyzed by Alcalase and Protamex (*p* < 0.05). Additionally, *R*. *hispidum* hydrolysates showed significantly greater reducing power than *N. nomurai* hydrolysates (*p* < 0.05). This result, consistent with data on O_2_•^−^ scavenging activity and molecular weight distribution, collectively indicates that hydrolysates with lower molecular weights possess stronger reducing and free radical scavenging capacities.

Using glutathione (GSH) as the positive control, the results shown in Figure 6 indicate that the DPPH• and O_2_•^−^ scavenging rates and the reducing power of the six samples were significantly lower than those of GSH. In contrast, their •OH scavenging activity was markedly higher (*p* < 0.05). These results suggest that jellyfish peptides possess selective antioxidant properties, which may be attributed to their abundance of hydrophilic amino acids such as Gly, Asp, and Glu. These residues can promote metal ion chelation and effectively remove hydroxyl radicals. Therefore, the primary antioxidant mechanism of these peptides is likely based on •OH scavenging rather than direct hydrogen donation. GSH, in comparison, is an efficient hydrogen donor and primarily exerts its antioxidant activity through hydrogen atom transfer.

## 3. Materials and Methods

### 3.1. Materials

Jellyfish *R. hispidum* and *N. nomurai* were purchased from Qingdao market, China. Flavourzyme (CAS: 9001-92-7) and Protamex (CAS: 9014-01-1) were obtained from Solarbio Co., Ltd., Beijing, China. Alcalase (EC: 3.4.21.14) was sourced from Shanghai yuanye Bio-Technology Co., Ltd. (Shanghai, China).

### 3.2. Jellyfish Enzymatic Hydrolysis Process

The hydrolysates were prepared according to the following procedure. First, jellyfish umbrellas were thoroughly cleaned with tap water to remove impurities. Subsequently, the cleaned umbrellas were homogenized to form a uniform mixture. The resulting homogenate was then subjected to enzymatic hydrolysis under different conditions (Table 5, Table 6 and Table 7). The enzymatic reaction was terminated by heating the mixture at 100 °C for 10 min. After cooling to room temperature, the hydrolysate was centrifuged at 10,380× *g* for 30 min at 4 °C to separate insoluble residues from the liquid fraction. The supernatant was carefully collected and concentrated using a rotary evaporator. Finally, the concentrate was lyophilized to obtain the jellyfish hydrolysate as a freeze-dried powder, which was stored at −20 °C for subsequent analysis.

### 3.3. The Ninhydrin Colorimetry Assay

Ninhydrin is reduced by the free α-amino groups, resulting in the formation of a blue-purple complex. Although ninhydrin assay is not a specific method for peptide content determination, the absorbance at 570 nm (A_570nm_) of the hydrolysates shows a linear correlation with the free α-amino groups concentration [29], and a higher value of A_570nm_ indicates a higher degree of hydrolysis, reflecting more thorough substrate breakdown. So, the A_570nm_ is commonly used as a robust and reliable metric for comparing the relative efficiency of the different enzymatic treatments. Consequently, the statistical analysis of A_570nm_ remains valid for identifying significant differences between experimental groups. A total of 980 μL deionized water was added to test tubes, followed by 20 μL hydrolysate and 500 μL ninhydrin chromogenic reagent. The mixture was then allowed to cool in an ice-water bath, followed by mixing and heating at 100 °C for 10 min. The absorbance was measured at 570 nm.

### 3.4. Single-Factor Experiments of Proteolysis

Based on our previous research [11], Alcalase, Flavourzyme and Protamex were selected for single-factor enzymatic hydrolysis. Five proteolysis parameters of three enzymes, namely enzyme dosage, hydrolysis time, temperature, pH and solid-liquid ratio were shown in Table 5. The A_570nm_ of ninhydrin colorimetry assay was used as the evaluation index.

### 3.5. Proteolysis Orthogonal Experiments

Single-factor experiments revealed that enzyme dosage, hydrolysis time, temperature, pH, and liquid-to-solid ratio all significantly influenced the hydrolysis outcomes for each of the three enzymes. Consequently, an orthogonal array design was employed to optimize these conditions, with each of the five factors set at three levels (Table 6). The experiments were conducted according to the orthogonal design matrix (Table 7) to determine the optimal hydrolysis conditions for each enzyme.

### 3.6. Determination of Degree of Hydrolysis (DH)

The degree of hydrolysis (DH) represents the percentage of cleaved peptide bonds, calculated from the amino peptide nitrogen content of the hydrolysis and the total nitrogen content of the substrate [30]. Samples were prepared using Alcalase, Flavourzyme, and Protamex under the conditions listed in Table 4. Amino peptide nitrogen content was determined according to GB 5009.235-2016. Total nitrogen content of the substrate was measured according to the Kjeldahl method (GB 5009.5-2016). DH was calculated according to Formula (1):

(1) $$\mathrm{DH}\;(\%)=\frac{h}{h_{tot}}\times 100\%$$

where

h: The amino peptide nitrogen content;

h_tot_: Total nitrogen content of the substrate.

### 3.7. Determination of Molecular Weight Distribution

The lyophilized jellyfish hydrolysate was dissolved in water to prepare a 10 mg/mL solution, filtered through a 0.22 μm aqueous membrane, and analyzed by high-performance liquid chromatography (HPLC) [31]. Samples were prepared using Alcalase, Flavourzyme, and Protamex under the hydrolysis conditions listed in Table 4. Mobile phase A was an aqueous solution containing 0.1% glacial acetic acid, and mobile phase B was acetonitrile. The ratio of A to B was 85:15, with the flow rate of the mobile phase controlled at 0.5 mL/min. The column temperature of the chromatographic column (TSKgel G2000SWXL, 7.8 × 300) was maintained at 25 °C, and the detector temperature was set to 25 °C. Ultraviolet absorption was measured at 215 nm, with an injection volume of 20 µL. Standard curves for peptide molecular weight were established using bovine insulin (5733.49 Da), thymalfasin (3108.275 Da), tetrapeptide (GGYR) (451.48 Da), L-tyrosine (247.25 Da), and glycyl-glycyl-glycine (189.17 Da) as standards. Retention time (Rt) was plotted against the logarithm of molecular weight (lg MW), with chromatographic peaks recorded. Retention times of hydrolysates were interpolated into the standard curve to calculate molecular weight ranges, and distribution profiles were quantified based on peak area integration.

### 3.8. Determination of Antioxidant Activity

Samples were prepared using Alcalase, Flavourzyme, and Protamex under the hydrolysis conditions listed in Table 4. The DPPH• scavenging activity of hydrolysates was determined according to the method described by Yu et al. [32]. Briefly, 1.5 mL hydrolysate (9 mg/mL) was mixed with 1.5 mL absolute ethanol, followed by addition of 1.0 mL of 0.1 mM DPPH ethanolic solution. After thorough vortexing and incubation for 30 min in darkness, absorbance was measured at 517 nm. The blank contained 50% ethanol, while the control group used 50% ethanol to replace the hydrolysate with 1.0 mL DPPH solution. A solution of GSH at the same concentration was used as a positive control. All tests were performed in triplicate. The DPPH• scavenging rate was calculated according to Formula (2):

(2) $$\mathrm{DPPH}•\;\mathrm{scavenging}\;\mathrm{rate}\;\left(\%\right)=\left[1-\frac{A_{1}-A_{2}}{A_{0}-A_{2}}\right]\times 100\%$$

where

A_0_ = absorbance of control group;

A_1_ = absorbance of test group;

A_2_ = absorbance of blank group.

The •OH scavenging activity was measured according to the method described by Yu et al. [32]. Briefly, 1 mL hydrolysate (9 mg/mL) was mixed sequentially with 1 mL PBS (150 mM, pH 7.4), 0.5 mL EDTA-Fe^2+^ solution (220 μM), 1 mL crocin (0.36 mg/mL in PBS), and 1 mL H_2_O_2_ (3% in PBS). After vortexing, the mixture was incubated at 37 °C for 30 min, then the absorbance at 520 nm was measured by spectrophotometry, deionized water instead of the hydrolysate as the blank and PBS instead of H_2_O_2_ as the control. A solution of GSH at the same concentration was used as a positive control. The •OH scavenging rate was calculated according to Formula (3):

(3) $$•\mathrm{OH}\;\mathrm{scavenging}\;\mathrm{rate}\;\left(\%\right)=\frac{A_{1}-A_{2}}{A_{0}-A_{2}}\times 100\%$$

where

A_0_ = absorbance of control group;

A_1_ = absorbance of test group;

A_2_ = absorbance of blank group.

The scavenging effect of jellyfish hydrolysates on O_2_•^−^ was assessed using the PMS-NADH-NBT system [33]. Sequentially add 0.5 mL of 78 μmol/L NADH, 0.5 mL of 300 μmol/L NBT, and 1.5 mL hydrolysate (9 mg/mL) into test tubes. Then add 0.5 mL of 90 μmol/L PMS to the mixture, vortex thoroughly, and incubate at room temperature for 5 min, the absorbance at 560 nm was measured by spectrophotometry. All assays were performed in triplicate, Tris-HCl buffer instead of the hydrolysate and NADH as the blank and Tris-HCl buffer instead of the hydrolysates as the control. A solution of GSH at the same concentration was used as a positive control. The O_2_•^−^ scavenging rate was calculated using Formula (4):

(4) $$O_{2}{•}^{-}\;\mathrm{scavenging}\;\mathrm{rate}\;(\%)=\frac{A_{0}-A_{1}}{A_{0}-A_{2}}\times 100\%$$

where

A_0_ = absorbance of control group;

A_1_ = absorbance of test group;

A_2_ = absorbance of blank group.

The reducing power of hydrolysates was measured according to the method described by Wang et al. [31]. Specifically, 0.5 mL hydrolysate (9 mg/mL), 2.5 mL PBS (0.2 mol/L, pH 6.6), and 2.5 mL potassium ferricyanide solution (1% *w/v*) were added into test tubes. After thorough mixing, the mixture was incubated at 50 °C for 30 min, then 2.5 mL trichloroacetic acid (10% *w/v*) was added to terminate the reaction. The mixture was allowed to stand at 25 °C for 10 min. Then, 2.5 mL of the standing solution was taken, followed by the addition of 2.5 mL of distilled water and 0.5 mL of 0.1% ferric chloride solution. After reacting at room temperature for 10 min, the absorbance of the reacted solution was measured at 700 nm by spectrophotometry. A solution of glutathione GSH at the same concentration was used as a positive control. The absorbance correlates positively with the reducing power of protein samples, with higher absorbance indicating stronger reducing power.

### 3.9. Statistical Analysis

All experiments were performed in triplicate, with data expressed as mean ± standard deviation (SD). Figures were generated using Origin 2024 (OriginLab, Northampton, MA, USA). Experimental data were analyzed with IBM SPSS 22.0 (IBM Corp., Armonk, NY, USA). One-way analysis of variance (ANOVA) was first performed to assess overall significance. This was followed by Duncan’s multiple range test as a post hoc procedure to determine specific differences between group means. Statistical significance was defined at *p* < 0.05.

## 4. Conclusions

This study highlighted the crucial role of molecular weight distribution in determining the antioxidant potential of jellyfish hydrolysates. Flavourzyme hydrolysates contained the highest proportion of low-molecular-weight peptides (<3 kDa), resulting in significantly stronger •OH and O_2_•^−^ scavenging activity and reducing power. In contrast, Protamex produced more high-molecular-weight fractions with weaker antioxidant effects. Moreover, *R. hispidum* hydrolysates exhibited superior DPPH• and O_2_•^−^ scavenging abilities compared to *N. nomurai* hydrolysates, likely due to their higher low-molecular-weight peptide content. These findings demonstrate that jellyfish species and enzymatic conditions determine peptide molecular weight and activity, and smaller peptides contribute more effectively to free radical scavenging. Future studies should identify the specific active peptide sequences and clarify their structure–activity relationships to better understand antioxidant mechanisms.

## Figures and Tables

**Table 1:** Results of orthogonal experiments of Alcalase.

Run Number	Time (A, h)	Liquid-to-Solid Ratio (B)	Temperature (C, °C)	Enzyme Dosage (D, U/g)	pH (E)	A_570nm_
1	4	6:1	50	2000	8.5	0.39
2	4	6:1	50	2000	9.0	0.37
3	4	6:1	50	2000	9.5	0.40
4	4	8:1	55	3000	8.5	0.26
5	4	8:1	55	3000	9.0	0.30
6	4	8:1	55	3000	9.5	0.32
7	4	10:1	60	4000	8.5	0.32
8	4	10:1	60	4000	9.0	0.33
9	4	10:1	60	4000	9.5	0.36
10	6	6:1	55	4000	8.5	0.30
11	6	6:1	55	4000	9.0	0.34
12	6	6:1	55	4000	9.5	0.38
13	6	8:1	60	2000	8.5	0.38
14	6	8:1	60	2000	9.0	0.39
15	6	8:1	60	2000	9.5	0.38
16	6	10:1	50	3000	8.5	0.28
17	6	10:1	50	3000	9.0	0.32
18	6	10:1	50	3000	9.5	0.33
19	8	6:1	60	3000	8.5	0.41
20	8	6:1	60	3000	9.0	0.40
21	8	6:1	60	3000	9.5	0.41
22	8	8:1	50	4000	8.5	0.36
23	8	8:1	50	4000	9.0	0.35
24	8	8:1	50	4000	9.5	0.35
25	8	10:1	55	2000	8.5	0.39
26	8	10:1	55	2000	9.0	0.38
27	8	10:1	55	2000	9.5	0.39
k_1_	3.05	3.39	3.15	3.46	3.10	
k_2_	3.11	3.10	3.07	3.04	3.19	
k_3_	3.44	3.10	3.38	3.10	3.32	
R	0.39	0.29	0.31	0.42	0.22	
Optimal Levels	A_3_	B_1_	C_3_	D_1_	E_3_	
*p*-value	<0.001	0.002	0.002	<0.001	0.037	

**Table 2:** Results of orthogonal experiments of Flavourzyme.

Run Number	Time (A, h)	Liquid-to-Solid Ratio (B)	Temperature (C, °C)	Enzyme Dosage (D, U/g)	pH (E)	A_570nm_
1	4	6	45	2000	6.5	0.61
2	4	6	45	2000	7.0	0.61
3	4	6	45	2000	7.5	0.61
4	4	8	50	3000	6.5	0.64
5	4	8	50	3000	7.0	0.65
6	4	8	50	3000	7.5	0.67
7	4	10	55	4000	6.5	0.81
8	4	10	55	4000	7.0	0.85
9	4	10	55	4000	7.5	0.86
10	6	6	50	4000	6.5	0.77
11	6	6	50	4000	7.0	0.80
12	6	6	50	4000	7.5	0.80
13	6	8	55	2000	6.5	0.64
14	6	8	55	2000	7.0	0.77
15	6	8	55	2000	7.5	0.66
16	6	10	45	3000	6.5	0.71
17	6	10	45	3000	7.0	0.73
18	6	10	45	3000	7.5	0.76
19	8	6	55	3000	6.5	0.71
20	8	6	55	3000	7.0	0.68
21	8	6	55	3000	7.5	0.65
22	8	8	45	4000	6.5	0.73
23	8	8	45	4000	7.0	0.74
24	8	8	45	4000	7.5	0.77
25	8	10	50	2000	6.5	0.71
26	8	10	50	2000	7.0	0.75
27	8	10	50	2000	7.5	0.79
k_1_	6.31	6.25	6.26	6.16	6.33	
k_2_	6.66	6.27	6.59	6.21	6.61	
k_3_	6.53	6.98	6.65	7.13	6.57	
R	0.35	0.73	0.39	0.98	0.28	
Optimal Levels	A_2_	B_3_	C_3_	D_3_	E_2_	
*p*-value	0.028	<0.001	0.006	<0.001	0.055	

**Table 3:** Results of orthogonal experiments of Protamex.

Run Number	Time (A, h)	Liquid-to-Solid Ratio (B)	Temperature (C, °C)	Enzyme Dosage (D, U/g)	pH (E)	A_570nm_
1	4	6	45	2000	6.0	0.35
2	4	6	45	2000	6.5	0.36
3	4	6	45	2000	7.0	0.36
4	4	8	50	3000	6.0	0.38
5	4	8	50	3000	6.5	0.39
6	4	8	50	3000	7.0	0.40
7	4	10	55	4000	6.0	0.41
8	4	10	55	4000	6.5	0.41
9	4	10	55	4000	7.0	0.43
10	6	6	50	4000	6.0	0.44
11	6	6	50	4000	6.5	0.47
12	6	6	50	4000	7.0	0.45
13	6	8	55	2000	6.0	0.38
14	6	8	55	2000	6.5	0.39
15	6	8	55	2000	7.0	0.39
16	6	10	45	3000	6.0	0.41
17	6	10	45	3000	6.5	0.42
18	6	10	45	3000	7.0	0.43
19	8	6	55	3000	6.0	0.42
20	8	6	55	3000	6.5	0.43
21	8	6	55	3000	7.0	0.47
22	8	8	45	4000	6.0	0.49
23	8	8	45	4000	6.5	0.49
24	8	8	45	4000	7.0	0.49
25	8	10	50	2000	6.0	0.44
26	8	10	50	2000	6.5	0.45
27	8	10	50	2000	7.0	0.44
k_1_	3.50	3.75	3.80	3.57	3.72	
k_2_	3.78	3.81	3.87	3.74	3.82	
k_3_	4.12	3.84	3.74	4.09	3.86	
R	0.62	0.08	0.13	0.53	0.14	
Optimal Levels	A_3_	B_3_	C_2_	D_3_	E_3_	
*p*-value	<0.001	0.121	0.011	<0.001	0.007	

**Table 4:** Optimal enzymatic hydrolysis conditions for individual enzymes.

Enzyme	Time (A, h)	Liquid-to-Solid Ratio (B)	Temperature (C, °C)	Enzyme Dosage (D, U/g)	pH (E)
Alcalase	8	6:1	60	2000	9.5
Flavourzyme	6	6:1	50	3000	7.0
Protamex	8	6:1	50	4000	7.0

**Table 5:** Experimental protocols for single-factor experiments.

Enzyme	Time (A,h)	Liquid-to-Solid Ratio (B)	Temperature (C, °C)	Enzyme Dosage (D, U/g)	pH (E)
Alcalase	2	2:1	40	1000	8.0
	4	4:1	45	2000	8.5
	6	6:1	50	3000	9.0
	8	8:1	55	4000	9.5
	10	10:1	60	5000	10.0
Flavourzyme	2	2:1	40	1000	6.0
	4	4:1	45	2000	6.5
	6	6:1	50	3000	7.0
	8	8:1	55	4000	7.5
	10	10:1	60	5000	8.0
Protamex	2	2:1	45	1000	5.5
	4	4:1	50	2000	6.0
	6	6:1	55	3000	6.5
	8	8:1	60	4000	7.0
	10	10:1	65	5000	7.5

**Table 6:** Factors and levels for orthogonal experiments.

Enzyme	Levels	Time (A, h)	Liquid-to-Solid Ratio (B)	Temperature (C, °C)	Enzyme Dosage (D, U/g)	pH (E)
Alcalase	1	4	6:1	50	2000	8.5
	2	6	8:1	55	3000	9.0
	3	8	10:1	60	4000	9.5
Flavourzyme	1	4	6:1	45	2000	6.5
	2	6	8:1	50	3000	7.0
	3	8	10:1	55	4000	7.5
Protamex	1	4	6:1	45	2000	6.0
	2	6	8:1	50	3000	6.5
	3	8	10:1	55	4000	7.0

**Table 7:** L_27_ (3^5^) orthogonal array design table.

Run Number	Time (A, h)	Liquid-to-Solid Ratio (B)	Temperature (C, °C)	Enzyme Dosage (D, U/g)	pH (E)
1	1	1	1	1	1
2	1	1	1	1	2
3	1	1	1	1	3
4	1	2	2	2	1
5	1	2	2	2	2
6	1	2	2	2	3
7	1	3	3	3	1
8	1	3	3	3	2
9	1	3	3	3	3
10	2	1	2	3	1
11	2	1	2	3	2
12	2	1	2	3	3
13	2	2	3	1	1
14	2	2	3	1	2
15	2	2	3	1	3
16	2	3	1	2	1
17	2	3	1	2	2
18	2	3	1	2	3
19	3	1	3	2	1
20	3	1	3	2	2
21	3	1	3	2	3
22	3	2	1	3	1
23	3	2	1	3	2
24	3	2	1	3	3
25	3	3	2	1	1
26	3	3	2	1	2
27	3	3	2	1	3

## Data Availability

The original contributions presented in this study are included in the article. Further inquiries can be directed to the corresponding author.

## Abbreviations

The following abbreviations are used in this manuscript:

*R. hispidum*	*Rhopilema hispidum*
*N. nomurai*	*Nemopilema nomurai*
DH	Degree of hydrolysis
•OH	Hydroxyl radical
DPPH•	1,1-diphenyl-2-picrylhydrazyl radical
O2•−	Superoxide anion
GSH	Glutathione

## References

[1] Zhang Q., Song C., Zhao J., Shi X., Sun M., Liu J., Fu Y., Jin W., Zhu B. (2018). Separation and Characterization of Antioxidative and Angiotensin Converting Enzyme Inhibitory Peptide from Jellyfish Gonad Hydrolysate. Molecules.

[2] Upata M., Siriwoharn T., Makkhun S., Yarnpakdee S., Regenstein J.M., Wangtueai S. (2022). Tyrosinase Inhibitory and Antioxidant Activity of Enzymatic Protein Hydrolysate from Jellyfish (*Lobonema smithii*). Foods.

[3] Prommasith P., Surayot U., Autsavapromporn N., Rod-in W., Rachtanapun P., Wangtueai S. (2024). Immunomodulatory, Anticancer, and Antioxidative Activities of Bioactive Peptide Fractions from Enzymatically Hydrolyzed White Jellyfish (*Lobonema smithii*). Foods.

[4] De Domenico S., De Rinaldis G., Paulmery M., Piraino S., Leone A. (2019). Barrel Jellyfish (*Rhizostoma pulmo*) as Source of Antioxidant Peptides. Mar. Drugs.

[5] Leone A., Lecci R.M., Durante M., Meli F., Piraino S. (2015). The Bright Side of Gelatinous Blooms: Nutraceutical Value and Antioxidant Properties of Three Mediterranean Jellyfish (Scyphozoa). Mar. Drugs.

[6] Muangrod P., Charoenchokpanich W., Roytrakul S., Rungsardthong V., Charoenlappanit S., Wonganu B., Tabtimmai L., Chamsodsai P., Casanova F., Thumthanaruk B. (2025). Bioactivity assessment of peptides derived from salted jellyfish (*Rhopilema hispidum*) byproducts. PLoS ONE.

[7] Felician F.F., Yu R.H., Li M.Z., Li C.J., Chen H.Q., Jiang Y., Tang T., Qi W.Y., Xu H.M. (2019). The wound healing potential of collagen peptides derived from the jellyfish *Rhopilema esculentum*. Chin. J. Traumatol..

[8] Barzkar N., Bunphueak P., Chamsodsai P., Muangrod P., Thumthanaruk B., Rungsardthong V., Tabtimmai L. (2024). Jellyfish protein hydrolysates: Multifunctional bioactivities unveiled in the battle against diabetes, inflammation, and bacterial pathogenesis. Microb. Pathog..

[9] Ballesteros A., Torres R., Pascual-Torner M., Revert-Ros F., Tena-Medialdea J., García-March J.R., Lloret J., Gili J.M. (2025). Jellyfish Collagen in the Mediterranean Spotlight: Transforming Challenges into Opportunities. Mar. Drugs.

[10] Killi N., Bonello G., Mariottini G.L., Pardini P., Pozzolini M., Cengiz S. (2020). Nematocyst types and venom effects of *Aurelia aurita* and *Velella velella* from the Mediterranean Sea. Toxicon.

[11] Teng L.C., Wang X.Q., Yu H.H., Li R.F., Geng H., Xing R.E., Liu S., Li P. (2023). Jellyfish Peptide as an Alternative Source of Antioxidant. Antioxidants.

[12] Leduc A., Fournier V., Henry J. (2020). A standardized, innovative method to characterize the structure of aquatic protein hydrolysates. Heliyon.

[13] Zhu H., Zhang Y., Yang T., Zheng D.Y., Liu X.J., Zhang J.T., Zheng M.M. (2022). Preparation of immobilized Alcalase based on metal affinity for efficient production of bioactive peptides. LWT-Food Sci. Technol..

[14] Zhang W.C., Chan J.X., Lu Y.Y., Liu S.Q. (2022). Pre-treatment of coconut kernels by proteases to modulate the flavour of coconut oil. Food Biosci..

[15] Zheng X.Q., Wang J.T., Liu X.L., Sun Y., Zheng Y.J., Wang X.J., Liu Y. (2015). Effect of hydrolysis time on the physicochemical and functional properties of corn glutelin by Protamex hydrolysis. Food Chem..

[16] Mongkonkamthorn N., Malila Y., Regenstein J.M., Wangtueai S. (2021). Enzymatic Hydrolysis Optimization for Preparation of Tuna Dark Meat Hydrolysate with Antioxidant and Angiotensin I-Converting Enzyme (ACE) Inhibitory Activities. J. Aquat. Food Prod. Technol..

[17] Qu J., Zhang M., Hong T., Xu X., Xu D. (2023). Improvement of adzuki bean paste quality by Flavourzyme-mediated enzymatic hydrolysis. Food Biosci..

[18] Lu X., Zhang L.X., Sun Q., Song G.H., Huang J.N. (2019). Extraction, identification and structure-activity relationship of antioxidant peptides from sesame (*Sesamum indicum* L.) protein hydrolysate. Food Res. Int..

[19] Landi N., Clemente A., Pedone P.V., Ragucci S., Di Maro A. (2022). An Updated Review of Bioactive Peptides from Mushrooms in a Well-Defined Molecular Weight Range. Toxins.

[20] Seong S.H., Lee Y.I., Lee J.H., Choi S., Kim I.A., Suk J., Jung I.H., Baeg C., Kim J., Oh D. (2024). Low-molecular-weight collagen peptides supplement promotes a healthy skin: A randomized, double-blinded, placebo-controlled study. J. Cosmet. Dermatol..

[21] Yu F.M., He K., Dong X.Z., Zhang Z.W., Wang F.L., Tang Y.P., Chen Y., Ding G.F. (2020). Immunomodulatory activity of low molecular-weight peptides from *Nibea japonica* skin in cyclophosphamide-induced immunosuppressed mice. J. Funct. Foods.

[22] Ballatore M.B., Bettiol M.D., Vanden Braber N.L., Aminahuel C.A., Rossi Y.E., Petroselli G., Erra-Balsells R., Cavaglieri L.R., Montenegro M.A. (2020). Antioxidant and cytoprotective effect of peptides produced by hydrolysis of whey protein concentrate with trypsin. Food Chem..

[23] Pezeshk S., Ojagh S.M., Rezaei M., Shabanpour B. (2019). Fractionation of Protein Hydrolysates of Fish Waste Using Membrane Ultrafiltration: Investigation of Antibacterial and Antioxidant Activities. Probiotics Antimicrob. Proteins.

[24] Gao J.J., Ning C., Wang M.X., Wei M.M., Ren Y.F., Li W.X. (2024). Structural, antioxidant activity, and stability studies of jellyfish collagen peptide-calcium chelates. Food Chem.-X.

[25] Di Filippo G., Melchior S., Plazzotta S., Calligaris S., Innocente N. (2024). Effect of enzymatic hydrolysis with Alcalase or Protamex on technological and antioxidant properties of whey protein hydrolysates. Food Res. Int..

[26] Lee J., Koo N., Min D.B. (2004). Reactive oxygen species, aging, and antioxidative nutraceuticals. Compr. Rev. Food Sci. Food Saf..

[27] Sun T., Xie W.M., Xu P.X. (2004). Superoxide anion scavenging activity of graft chitosan derivatives. Carbohydr. Polym..

[28] Canabady-Rochelle L.L.S., Harscoat-Schiavo C., Kessler V., Aymes A., Fournier F., Girardet J.M. (2015). Determination of reducing power and metal chelating ability of antioxidant peptides: Revisited methods. Food Chem..

[29] Wang S., Wang D., Tao L., Xu Y. (2019). Comparative study on methods for determination of amino acid nitrogen in Huangjiu and Mijiu. Food Ferment. Ind..

[30] Rutherfurd S.M. (2010). Methodology for Determining Degree of Hydrolysis of Proteins in Hydrolysates: A Review. J. AOAC Int..

[31] Wang X., Yu H., Xing R., Chen X., Liu S., Li P. (2018). Optimization of antioxidative peptides from mackerel (*Pneumatophorus japonicus*) viscera. PeerJ.

[32] Yu H.H., Liu X.G., Xing R.E., Liu S., Li C., Li P.C. (2005). Radical scavenging activity of protein from tentacles of jellyfish *Rhopilema esculentum*. Bioorg. Med. Chem. Lett..

[33] Wang L.Q., Guo R., Liang X.R., Ji Y.T., Zhang J.J., Gai G.W., Guo Z.Y. (2023). Preparation and Antioxidant Activity of New Carboxymethyl Chitosan Derivatives Bearing Quinoline Groups. Mar. Drugs.

